# Immunogenetic Markers Definition in Latvian Patients with Lyme Borreliosis and Lyme Neuroborreliosis

**DOI:** 10.3390/ijerph13121194

**Published:** 2016-12-01

**Authors:** Lilija Kovalchuka, Svetlana Cvetkova, Julija Trofimova, Jelena Eglite, Sandra Gintere, Irina Lucenko, Barbara Oczko-Grzesik, Ludmila Viksna, Angelika Krumina

**Affiliations:** 1 Institute of Food Safety, Animal Health and Environment BIOR, Riga LV-1076, Latvia; Svetlana.Cvetkova@bior.lv (S.C.); Julija.Trofimova@bior.lv (J.T.); Krumina.Angelika@inbox.lv (A.K.); 2 Laboratory of Clinical Immunology and Immunogenetic, Riga Stradiņš University, Riga LV-1067, Latvia; Jelena.Eglite@rsu.lv; 3 Department of Family Medicine, Riga Stradiņš University, Riga LV-1067, Latvia; Sandra.Gintere@rsu.lv; 4 Centre for Disease Prevention and Control of Latvia, Riga LV-1005, Latvia; Irina.lucenko@spkc.gov.lv; 5 Department of Infectious Diseases, Medical University of Silesia, 40-055 Katowice, Poland; bgrzesik@hoga.pl; 6 Department of Infectology and Dermatology, Riga Stradiņš University, Riga LV-1006, Latvia; Ludmila.Viksna@rsu.lv

**Keywords:** *Borrelia burgdorferi*, Lyme borreliosis, Lyme neuroborreliosis, immunogenetic markers, HLA-DRB1

## Abstract

The aim of this study was to determine the human leukocyte antigen (HLA)-DRB1 alleles in two groups of patients in Latvia: patients with Lyme borreliosis and patients with Lyme neuroborreliosis. The study included 216 patients with Lyme borreliosis, 29 patients with Lyme neuroborreliosis and 282 control persons. All surveyed persons were residents of Latvia. The HLA-DR genotyping was performed by polymerase chain reaction- sequence specific primer (PCR-SSP). The predisposition to the Lyme borreliosis is associated with the HLA-DRB1*07, -DRB1*17(03), -DRB1*04, -DRB1*15(02) alleles. The allele -DRB1*11(05), -DRB1*14(06) and -DRB1*13(06) were significantly more frequent in controls. In-group with Lyme neuroborreliosis differences were found for the -DRB1*07 and -DRB1*04 alleles, but only HLA-DRB1*07 allele was statistically significant after Bonferroni correction and associated with Lyme neuroborreliosis in Latvian patients.

## 1. Introduction

Lyme disease (LD, Lyme borreliosis) is the most prevalent vector-borne disease in Europe and the United States [1]. At present LD is considered as a natural foci, infectious, multisystem disease with complex pathogenesis, including a complex of immune-mediated reactions [2].

Different clinical manifestations of Lyme disease were known long time ago and were described as independent illnesses, or as syndromes of unclear etiology: chronic erythema migrans, erythema Afcelius, tick-borne erythema annulare, acrodermatitis, chronic athrophic acrodermatitis, lymphadenosis of the skin, serous meningitis, radiculoneuritis, Banwarth’s syndrome, or chronic arthritis. In 1981 the spirochetal etiology of these manifestations was established, which led to the conception of the disease as a nosological form of different clinical manifestations [3].

Until recent time, the causative agent of Lyme disease was considered to be a single borrelia species—*Borrelia burgdorferi*. However, some differences in protein content of Borrelia isolates from different natural foci allowed us to suggest that by etiology Lyme-borreliosis is heterogeneous [4,5,6]. At present 10 genome groups have been identified, relating to the complex *Borrelia burgdorferi sensu lato*, which are unevenly distributed along the globe [7,8,9]. In Europe and Asia there have been identified groups such as *В. burgdorferi sensu stricto*, *В. garinii*, *В. garinii* (type NT29), *В. afzelii*, *В. valaisiana* (group VS116), *В. lusitaniae* (group PotiB2), *B. japonica*, *В. tanukii* and *В. turdae*, but in Аmerica, the groups *B. burgdorferi s.s.*, *B. andersonii* (group DN127), 21038, CA55 and 25015 have been found. As to the discovery of *B. japonica* in Japan, it is, apparently, nonpathogenic for humans. It is worth mentioning, that at present, the pathogenic potential of the group VS116 (*В. valaisiana*) is also not clear.

### 1.1. Impact of Borrelia Diversity on Pathogenicity and Clinical Symptoms

Results of different studies and clinical observations of the last years allow us to suggest that the characteristics of organ damage of a patient depend on the species of Borrelia [10,11]. The data have been obtained by finding an association between *B. garinii* and neurological exposures, *B. burgdorferi s.s.* and Lyme-arthritis, and *B. afzelii* and chronic atrophic dermatitis. Thus, the observed differences in the clinical picture of Lyme disease show that the patient may have basically genetic heterogeneity of the complex *B. burgdorferi sensu lato* in different nosoareal sites of this infection [12].

### 1.2. The Vector (Ixodes)

Lyme borreliosis is transmitted by ticks of the *Ixodes ricinus* complex. Considering these facts, the term “Lyme Disease” is currently understood as a whole group of etiologically independent *Ixodes* tick-borne borreliosis. In Europe, the principal vector is *I. ricinus*, and in Asia it is *I. persulcatus* [13,14,15]. Notably, both species of *Ixodes* are common in Latvia.

In 1986 the first case of Lyme borreliosis was registered in Latvia. According to the official statistics, around 300 cases of LB are logged every year (Table 1) [16,17,18]. The largest number of cases was registered in 2010. Moreover, most of the cases of this disease are registered through epidemiological history (attached tick) and presence of clinical signs (erythema migrans), since other means of tick borreliosis diagnosis are still not advanced. This, undoubtedly, does not reflect the real amount of tick borreliosis infections in the country, where cases of Lyme disease have been recorded in all four regions (Kurzeme, Vidzeme, Zemgale, and Latgale) [16,18].

*Ixodes ricinus*, which can transfer several pathogens simultaneously, is the most widespread tick in Europe [19,20,21,22,23,24]. In Poland, a neighboring country, an increased incidence of Lyme has been also observed over the last decade (Table 1) [20]. The number of people infected in 2009 (8.783 persons) had increased by almost 58% in 2015 (up to 13,870 people), which is higher, according to statistics, than in other European countries.

It is assumed that climate change, with milder winters and a longer growing season, has contributed to the increased incidence in endemic areas over the past three decades [21,22,23,24,25]. Ticks have a long life cycle, including three active life stages (larva, nymph, and adult). In addition, they are able to feed themselves on more than 300 different vertebrate species [24,26]. Moreover, populations of large animals such as deer and wild boar are more numerous in suburban areas around cities in Europe, which has led to the development of populations of mites shifting the natural cycles of transmission of certain pathogens, and, as a consequence, to an increased risk of disease in humans in many European countries [24,25,26,27].

Lyme borreliosis present a serious danger to the human health, because it may result in long-term inability to work and disability. Currently, Lyme disease pathogenesis is not fully understood. The unpredictable character of the disease course, possible severity and duration of disease that determines the further quality of life for the individual, raises concerns. Nevertheless, the question remains: what are the conditions in the process of interaction between micro-and macroorganism set the course of the disease, severity, outcome and complications? The possibility of serious chronic forms of the disease, of course, depends not only on the properties of a virus infection, but also on the genetically determined host reactivity. Because Human Leukocyte Antigen (HLA) molecules are the products of genes of the immune response, significant polymorphisms in these genes may lead to differences in susceptibility and (or) resistance to infection. The aim of this study was to identify disease-associated HLA-DRB1 alleles in Latvian patients with Lyme borreliosis and Lyme neuroborreliosis. 

## 2. Materials and Methods

### 2.1. Сharacteristics of Persons Participating in the Study

The study included 216 patients with an established diagnosis of Lyme borreliosis and 282 healthy (control) persons. The included patients’ ages ranged from 18 to 68 years. The majority of patients were between 32 and 55 years of age, representing 60.4% of the total studied. The clinical diagnosis was confirmed at the Infectology Center of Latvia. Diagnoses were based on two-step serum immunoglobulin (Ig)M and IgG testing using enzyme-linked immunosorbent assay (ELISA) and immunoblots. In suspected cases of neuroborreliosis, cerebrospinal fluid assays were performed with estimation of *B. burgdorferi*-specific intrathecal antibody production. The control group consisted of blood donors aged from 18 to 65 years. Most of them, about 65.9%, were 20–35 years of age. According to the European rules on donation, they had been tested in the Riga State donor center. All patients and healthy persons were residents of Latvia.

All subjects gave their informed consent for inclusion before they participated in the study. The study was conducted in accordance with the Declaration of Helsinki, and the protocol was approved by the Ethics Committee of the Riga Stradins University under the number 2012-05-E9-2. The approval was signed by the Chairman of the Committee Dr. Olafs Brūvers and seven members of the committee 31.05.2012.

### 2.2. HLA Typing

Immunogenetic examinations were performed at the Laboratory of Clinical Immunology and Immunogenetics, Riga Stradinš University. Blood samples (2.5 mL) were collected from the subjects in tubes containing anticoagulant (EDTA) and centrifuged (“Hettich zentrifugen”, mikro 22 R, Tuttlingen, Germany) at 2500 rpm for 15 min, and the Buffy-coat was conserved at −20 °C until use. The genomic DNA was extracted from proteinase-K-treated peripheral blood leukocytes using the routine “salting-out” method [28]. The DNA was stored in tris-EDTA (TE) buffer (10 mL Tris-HCl, pH 7.5, and 2 mL 0.5 M Na2 EDTA per litter of distilled water). The DNA concentration, around 100–200 µg/mL was determined by fluorescence with a DNA fluorimeter.

HLA-DR genotyping by polymerase chain reaction (PCR) with sequence-specific primers (PCR–SSP) for DRB1*01 to DRB1*10 was performed [28,29,30]. The reaction mixture (15 µL) included 1.0 µL DNA, 1.5 µL PCR buffer (50 mM KCl, 1.5 mM MgCl_2_, 10 mM Tris-HCl (pH 8.3)), 0.6 µL dNTPs (25 mmol/L), 1.0 µL specific primers (0.2 mmol/L), and 0.5 U of the *Taq* DNA polymerase (Promega, Madison, WI, USA). The reaction mixture was subjected to 45 amplification cycles, each consisting of denaturation cycle at 94 °C (60 s), annealing cycles at 67 °C (40 s), and extension cycles at 65 °C (10 s). PCR products were visualized by agarose-gel electrophoresis [29,30]. After addition of 2 M loading buffer, the PCR reaction mixtures were loaded in agarose gels pre-stained with ethidium bromide (0.5 µL/mL gel). Gels were run for 15 min at 10 V/cm gel in 0.5 mM TBE (0.89 M Tris, 0.89 M boric acid and 0.02 M EDTA in aqueous solution) buffer and then were examined under ultraviolet (UV) illumination and recorded [29,30].

### 2.3. Statistical Analysis

The significance of differences in individual subtypes between patients and controls were performed using the Chi square test, with the Bonferroni correction, or Fisher’s Exact Test when necessary [31]. Application of Mantel-Haenszel or Yates correction was performed for statistical assessment of the heterogeneity in risk. Data were considered statistically significant when *p* value was less than or equal to 0.05. Moreover, to account for multiple comparisons, the observed *p* values were corrected (*pc*) for the number of alleles when locus was considered alone, thereby increasing the overall stringency of the significance testing. The odds ratios (OR), with 95% confidence intervals (95% CI), were calculated using the Simple Interactive Statistical Analysis (SISA) statistics online (http://home.clara.net/sisa/) website, to evaluate the risk of the individual developing the disease while having a particular HLA type.

## 3. Results

### 3.1. Clinical Characteristics of the Patient Group Participating in the Study

Traditionally, all patients were divided into three groups. The first group consisted of patients with stage I of the early period of the disease, when the pathogen gets into the skin after the tick bite (Table 2).

Incubation period was 1–32 days (on average 12 days). The stage was characterized by the development of a complex of inflammatory-allergic changes of the skin in the area of the tick bite, exposed in a specific way, characteristic for Lyme borreliosis-like skin manifestations—erythema (erythema migrans, EM). Erythema migrans was not observed in 16 persons (about 13.6% of the patients). Benign lymphoma of the skin is considered as one of the few clinical manifestations of Lyme’s disease, together with ring-type EM, characterized by a single infiltrate, or dissemination of platelets. Subjects experienced a rather satisfying wellness, showing a weak syndrome of general intoxication. Part of the patients complained of flu-like symptoms, fever, myalgia and arthralgia. In some cases conjunctivitis was observed.

The second group consisted of patients with stage II of the early period, when the introduction of Borrelia in various organs happens. This stage is characterized by high clinical polymorphisms with the possibility of Borrelia to enter into the organs and tissues and cause mono- and polyorgan damage. At the pathogen dissemination stage it was possible to identify the prevalent group of symptoms—neurological, cardiac, mixed—which determines the variant of the clinical process. Specifying the variant of the course of the disease helps us to define the severity of the pathologic process. Time of exposure—1 to 3 (sometimes up to 6) months. Damage of skeletomuscular apparatus was observed, such as arthralgia, tendonitis, bursitis, myalgia, and ostealgia, short attacks of recurrent arthritis, myositis, and panniculitis in 12.2% of the patients, which equals the incidence of *B. burgdorferi sensu stricto*.

The signs of the damage to the central and peripheral nervous system were observed, such as meningitis, mono- or polyneuritis, cranial-cerebral neuritis, quite often—facial nerve neuritis, movement radiculoneuritis, myeloradiculitis in 25.5% of the patients. All these syndromes can be observed in combinations.

Damage to the cardiovascular system was observed more rarely than the damage to the nervous system, and it does not show any characteristic features. The incidence of the damage to the cardio-vascular system made up 8.9% of the whole group (in Europe 0.2%–8.0%) [4,32]. Quite commonly, skin conditions and neurological symptoms were observed in parallel in some patients. Less common manifestations of borreliosis infection were eye damage: conjunctivitis, eye myositis, keratitis, inflammation of the optical nerve.

The third group included eight patients (3.7% of the total group) with the late disseminated disease stage and incubation periods of more than 6 months. Six persons from the group were diagnosed with neuroborreliosis, mainly, with mono-or polyneuritis, or neuritis of the facial nerve, and two persons with mixed pathologies.

The division of the disease into stages is relative, and can be applied to the disease, if only considered as a whole. Sometimes no stages may be observed at all, in some cases there may be only I stage, but sometimes the disease is exposed by one of the late stage symptoms (Table 2).

### 3.2. Immunogenetic Examinations of Group with Lyme Borreliosis

Our next step was to identify HLA-DRB1 alleles in two groups of patients: in the total group with LB (Table 3) and the group with LNB (Table 4). Earlier, we have described a DR association in Latvian patients with LB [17], but the study group was not big enough. For the genetic correlations is important that a sufficiently large number of people be included in the study. With the series of 216 patients with LB, the present study is the largest tested to date, while Latvian patients with neuroborreliosis are described for the first time.

Typing of all fourteen alleles DRB1were investigated. The frequency of DRB1* alleles in Latvian patients with Lyme borreliosis and control group are shown in Table 3. The predisposition to the Lyme disease is associated with the HLA-DRB1*07 (odds ratio 1.70; *p* = 0.021), HLA-DRB1*17(03) (odds ratio 1.55; *p* = 0.046), HLA-DRB1*04 (odds ratio 1.55; *p* = 0.046) and HLA-DRB1*15(02) (odds ratio 1.54; *p* = 0.042) alleles (Table 3). Although, these three differences were no longer significant when the pc value was corrected for the number of alleles (Table 3). 

Moreover, the allele -DRB1*11 (odds ratio 0.38; *p* = 0.0004), -DRB1*14 (odds ratio 0.51; *p* = 0.048) and -DRB1*13 (odds ratio 0.55; *p* = 0.025) was rarer in borreliosis patients and significantly more frequent in controls. For -DRB1*14 and -DRB1*13 alleles this detected HLA-DRB1 differences were not significant after Bonferroni correction (Table 3). Probably, only HLA-DRB1*11 allele (9.8 percent vs. 3.9 percent; odds ratio 0.38; *pc* = 0.006) has a protective effect in the pathogenesis of Lyme disease.

### 3.3. Immunogenetic Examinations of Group with Lyme Neuroborreliosis

Patients with LNB were examined separately. We analyzed a group with LNB, as the majority of Latvian patients have neurological complications (Table 2). The group included 29 persons (23 patients with stage II plus six patients with stage III). All patients had signs of damage to the central and peripheral nervous system. 25.5% expressed painful meningoradiculitis (with inflammation of the nerve roots), quite often—neuritis of the facial nerve, mono- or polyneuritis, often—meningitis, traumatic brain neuritis, and motion radiculoneuritis. All these syndromes can be observed in combination. The distribution of HLA-DRB1* alleles in LNB patients is presented in Table 4.

In the group of patients with the greatest frequency HLA-DRB1*07 and -DRB1*04 alleles (17.2 percent versus 6.6 percent; odds ratio 2.97; *p* = 0.003; and 15.5 percent against 7.4 percent; odds ratio 2.28; *p* = 0.033, respectively) were revealed. As well, the frequency of HLA-DRB1*15 allele (odds ratio 2.07; *p* = 0.060) was higher in LNB patients compared with the controls (Table 4). Despite the fact that the odds ratio in three detected alleles is high enough, the effect of the DRB1*04 and DRB1*15 alleles is controversial, because the *pс* value was larger than 0.05 (Table 4).

Only the HLA-DRB1*07 allele was statistically significant after applying the Bonferroni correction associated with Lyme neuroborreliosis in Latvian patients (odds ratio 2.97; *pc* = 0.041) (Table 4). Otherwise, the frequencies of HLA-DRB1*14 (06) and -DRB1*11(05) (odds ratio 0.31; *p* = 0.193; and, odds ratio 0.33; *p* = 0.113, respectively) were lower in LNB patients (Table 3). This data suggest that HLA-DRB1 molecules may have a considerable effect on susceptibility/or protection to Lyme neuroborreliosis.

## 4. Discussion

One of the most important currently unsolved problems is the study of peculiarities of interrelationship of the body and pathogenic genotypes of *Burgdorferi s.l.* [32,33]. Due to this, special interest is paid to the analysis of one of the basic systems of the body, controlling the immune response, HLA, including identification of possible associations with HLA genotypes of clinical features of Lyme disease.

Genetic investigations, conducted with adult patients with Lyme-caused arthritis, showed that molecules of the major histocompatibility complex (MHC) class II—DR2 and DR4, as well the association with anti-OspA-antibodies suggest a chronic course of arthritis and facilitate antibacterial therapy of low efficacy [34,35,36,37,38,39].

Most of the authors have described microbiological, and genetic characteristics of the very causative agent, and also the questions of pathophysiology of Ixodes tick borreliosis in a more detailed way. The research data are based on the results of experimental biochemical, patho-physiological and molecular-genetic methods of research using visualization methods [40,41,42,43]. In the future it will be important to broaden the diagnostic research, considering the regional peculiarities in the development of *Burgdorferi s.l.*, directed at clarifying pathogenetic mechanisms for the development of Ixodes tick borrelioses, which will help in studying the clinical characteristics and improving the tactics of therapeutic-preventive measures in the mentioned group of diseases.

A special place in studying the immunopathogenesis of Lyme Borreliosis is taken by neuroborreliosis [44,45,46,47,48]. Considering the immunological privilege of the nervous system and suggesting that the high level of proinflammatory cytokines can be the cause of prolonged location of Borrelia in tissues, and in the nervous system too [46,49,50], we can assume that these factors determine the development of chronic neuropathology. Earlier pathogenetic peculiarities of Lyme Borreliosis were described at the early infection stage, associated mainly with a certain amount of viable Borrelia in the inflammation site, while in the late stages the autoimmune mechanisms could contribute to the development of the disease. Long-term exposure of the immune system of the human host to the influence of *B. burgdorferi s.l.* is known to induce chronic autoimmune diseases [38]. Molecular mimicry is one of the autoimmune mechanisms, provoked by the infection, and can be related to the development of arthritis and myocarditis in case of Lyme disease. Triggering of autoimmune mechanisms, directly or indirectly mediated by *B. burgdorferi s.l.* on the basis of molecular mimicry is called one of the possible causes of antibiotic-refractory Lyme disease [51].

Alaedini and Latov, investigating the neurological manifestations of the chronic course of Lyme disease, including encephalopathy, myelopathy and peripheral neuropathies, have confirmed the role of autoimmune mechanisms in the development of the disease [52]. They exposed identical sequences of protein OspA (in DNA context), and DNA, isolated from brain tissues of mammalians. Then, taking a human as an example, it was proved that antibodies to two homologous peptides OspA react with neurons in the brain, spinal cord and spinal ganglia, causing the development of autoimmune inflammation in nerve tissue [52,53].

There are only a few data in the scientific literature about the interrelationship between neurological forms of borreliosis with single loci HLA II class. According to the studies on Latvian patients, genetic predisposition plays an essential role in the development of neuroborreliosis, determined by the vector of specific alleles DRB1 loci of HLA system. According to the obtained data, the alleles DRB1*07 and DRB1*04 are risk factors for the development of neuroborreliosis in Latvian patients, while the allele DRB1*07 is prognostically unfavorable from the point of view of the development of severe forms of the disease. Nevertheless, allele DRB1*11 possesses the definite protective factor. Thus, our data provide a possible explanation for the differential regulation of the immune response in patients with DRB1*07, DRB1*04 and DRB1*11 upon Bb-infection; namely, HLA-DRB1*07, and -DRB1*04 would predispose individuals to chronic disease development by generating an inflammatory milieu for Borrelia, while HLA-DRB1*11 would exert a protective role through the production of anti-spirochetal antibodies, which bind to the bacteria and eliminate it.

In such a way, despite the 30-year long experience of studying the peculiarities of the relationship between the causative agent LB, *Borrelia burgdorferi s.l.*, tick transmitters and the host’s body, it shows that our knowledge about the pathogenesis of the mentioned infection remains insufficient, as it does not allow us to understand the essence of the pathologic process in all its details. Therefore, it is necessary to continue research and extend the range of persons under investigation.

## 5. Conclusions

The risk of developing a chronic form of borreliosis infection is connected with clinical manifestations of the acute period of the disease, the presence of specific risk markers, and also with the timely and adequate choice of the antibiotic used. During the early stages of Lyme’s disease antibiotic therapy is well tolerated, whereas during the later stages the therapy takes longer and is not always successful.

Genotyping of HLA-DRB1 alleles at the very start of the disease can produce valuable prognosis in the development of the disease and the choice of the most optimal therapy. According to the results, the risk alleles for Latvian LB patients are the HLA-DRB1*07, -DRB1*17(03), -DRB1*04 and -DRB1*15(02) alleles, whereas, the development of severe forms of neuroborreliosis is strongly associated with the HLA-DRB1*07 allele. In contrast, the HLA-DRB1*11 allele has a certain protective effect in the pathogenesis of LB. The data obtained may find use in clinical practice for prediction of possible severe disease form development in patients with a certain HLA-genotype.

## Figures and Tables

**Table 1:** Number of Borreliosis cases, per 100,000 inhabitants in Latvia and Poland.

Years	2010	2011	2012	2013	2014	2015
Latvia, per 100,000 inhabitants	39.5	38.9	35.6	22.4	23.5	24.9
Poland, per 100,000 inhabitants	23.6	23.8	22.8	33.1	36.0	35.4

Source: Centre of Disease Prevention and Control (CDPC) of Latvia (Latvia) [18]; “Choroby zakaźne w Polsce”, PZH for years 2010–2015, LD (Poland) [20].

**Table 2:** Distribution of clinical borreliosis manifestations in patients from Latvia.

Early Localized Disease/Stage I (<30 Days), *n* = 118			* Early Disseminated Disease/Stage II (>30 Days), *n* = 90			Late Disseminated Disease/Stage III (>6 Months), *n* = 8	
	%	*n*		%	*n*		*n*
EM (-)	13.6	16	EM (-)	37.8	34		
			LNB	25.5	23	LNB	6
			Cardiac LB	08.9	08		
			LA	12.2	11		
			Mix/others	46.7	48	Mix/others	2

Abbreviations: EM (-): nonerythematous form; LNB: Lyme neuroborreliosis; LB: Lyme borreliosis; LA: Lyme arthritis; * the groups with prevalent symptoms.

**Table 3:** The frequency of DRB1* alleles studied in-patients with Lyme borreliosis and healthy controls from Latvia.

Allele HLA-	LB Patients (*n* = 216)		Controls (*n* = 282)		OR	(95% CI)	*p*-Value	*pc*-Value	χ^2^
	%	*N*	%	*N*					
DRB1*01	10.9	47	12.6	71	0.85	0.56–1.28	0.408	ND	0.68 M
DRB1*15(02)	12	52	8.2	46	**1.54**	0.99–2.39	**0.042**	0.452	3.73 Y
DRB1*16(02)	10.9	47	10.8	61	1.01	0.66–1.53	0.974	ND	0.00
DRB1*17(03)	11.1	48	7.4	42	**1.55**	0.98–2.45	**0.046**	0.483	3.99 M
DRB1*18(03)	8.1	35	5.1	29	1.63	0.95–2.79	0.059	ND	3.56 M
DRB1*04	11.1	48	7.4	42	**1.55**	0.98–2.45	**0.046**	0.483	3.99 M
DRB1*11(05)	3.9	17	9.8	55	**0.38**	0.21–0.68	**0.0004**	**0.006**	11.49 Y
DRB1*12(05)	4.2	18	5.5	31	0.75	0.40–1.40	0.336	ND	0.66 Y
DRB1*13(06)	4.9	21	8.5	48	**0.55**	0.31–0.96	**0.025**	0.298	4.50 Y
DRB1*14(06)	2.8	12	5.3	30	**0.51**	0.24–1.05	**0.048**	0.498	3.91 M
DRB1*07	10.6	46	6.6	37	**1.70**	1.06–2.73	**0.021**	0.257	5.35 M
DRB1*08	2.8	12	3.7	21	0.74	0.34–1.59	0.409	ND	0.42 Y
DRB1*09	2.8	12	3.4	19	0.82	0.37–1.80	0.595	ND	0.12 Y
DRB1*10	2.3	10	4.1	23	0.56	0.24–1.24	0.124	ND	1.86 Y
DRB1*x	1.6	7	1.6	9	ND	ND	ND	ND	ND

The bold alleles are statistically significant (*p* < 0.05). Abbreviations: ND: not defined; OR: odds ratio; CI: confidence interval; *p*-value (probability); *pc*-value (probability after Bonferroni adjustment); M: Mantel-Haenszel; Y: Yates corrected; χ^2^: Chi-Squares test.

**Table 4:** The frequency of DRB1* alleles studied in-patients with Lyme neuroborreliosis and healthy controls from Latvia.

Allele HLA-	LNB Patients (*n* = 29)		Controls (*n* = 282)		OR	(95% CI)	*p*-Value	*pc*-Value	χ^2^
	%	*N*	%	*N*					
DRB1*01	5.2	3	12.6	71	0.38	0.09–1.30	0.096	ND	2.76 M
DRB1*15(02)	15.5	9	8.2	46	2.07	0.88–4.71	0.060	ND	2.68 Y
DRB1*16(02)	6.9	4	10.8	61	0.61	0.18–1.84	0.352	ND	0.86 M
DRB1*17(03)	5.2	3	7.4	42	0.68	0.16–2.38	0.377	ND	0.40 M
DRB1*18(03)	5.2	3	5.1	29	1.01	0.24–3.61	0.589	ND	0.09 M
DRB1*04	15.5	9	7.4	42	**2.28**	0.97–5.23	**0.033**	0.375	3.54 Y
DRB1*11(05)	3.4	2	9.8	55	0.33	0.05–1.43	0.113	ND	1.81 Y
DRB1*12(05)	3.4	2	5.5	31	0.61	0.10–2.73	0.388	ND	0.44 M
DRB1*13(06)	5.2	3	8.5	48	0.59	0.14–2.04	0.276	ND	0.78 M
DRB1*14(06)	1.7	1	5.3	30	0.31	0.02–2.20	0.193	ND	1.43 M
DRB1*07	17.2	10	6.6	37	**2.97**	1.29–6.67	**0.003**	**0.041**	8.58 M
DRB1*08	6.9	4	3.7	21	1.88	0.53–6.08	0.204	ND	0.29 M
DRB1*09	5.2	3	3.4	19	1.56	0.36–5.83	0.337	ND	0.50 M
DRB1*10	1.7	1	4.1	23	0.41	0.02–2.95	0.325	ND	0.78 M
DRB1*x	5.2	3	1.6	9	ND	ND	ND	ND	ND

The bold alleles are statistically significant (*p* < 0.05). Abbreviations: ND: not defined; *p*-value (probability); *pc*-value (probability after Bonferroni adjustment); M: Mantel-Haenszel; Y: Yates corrected; χ^2^: Chi-Squares test.
